# Development of sleep patterns in children with obese and normal‐weight parents

**DOI:** 10.1111/jpc.14294

**Published:** 2018-11-10

**Authors:** Lijuan Xiu, Maria Hagströmer, Linnea Bergqvist‐Norén, Elin Johansson, Kerstin Ekbom, Viktoria Svensson, Claude Marcus, Mirjam Ekstedt

**Affiliations:** ^1^ Division of Pediatrics, Department of Clinical Science Intervention and Technology, Karolinska Institutet Stockholm Sweden; ^2^ Division of Physiotherapy, Department of Neurobiology, Care Sciences and Society Karolinska Institutet Stockholm Sweden; ^3^ Department of Learning, Informatics, Management and Ethics Medical Management Centre, Karolinska Institutet Stockholm Sweden; ^4^ Faculty of Health and Life Sciences, Linnaeus University Kalmar Sweden

**Keywords:** child sleep, childhood obesity, sleep variation

## Abstract

**Aim:**

To study the sleep development and sleep characteristics in children at different obesity risks, based on parental weight, and also to explore their weekday–weekend sleep variations and associated family factors.

**Methods:**

A total of 145 children participating in a longitudinal obesity prevention project were included, of which 37 had normal‐weight parents (low obesity risk), and 108 had overweight/obese parents (high obesity risk). Sleep diaries at ages 1 and 2 years were used to study sleep development in children at different obesity risks. Objectively assessed sleep using an accelerometer at 2 years of age was used to analyse weekday–weekend sleep variations.

**Results:**

There was no difference in sleep development from age 1 to age 2 among children at different obesity risks, but more children in the high‐risk group had prolonged sleep onset latency and low sleep efficiency. At 2 years of age, children in the high‐risk group had more weekday–weekend variation in sleep offset (mean difference 18 min, 95% confidence interval (CI) 4–33 min), midpoint of sleep (mean difference 14 min, 95% CI 3–25 min) and nap onset (mean difference 42 min, 95% CI 10–74 min) than children in the low‐risk group, after adjusting for other family factors. However, no difference could be detected between groups in weekday–weekend variation in sleep duration.

**Conclusions:**

Unfavourable sleep characteristics, as well as more variation in sleep schedules, have been observed in children at high obesity risk. While the differences were relatively small, they may reflect the unfavourable sleep hygiene in families at high obesity risk.

## What is already known on this topic

1. Obesity in parents has been identified as a major risk factor for child obesity; the transfer of adiposity through generations is probably not only related to genetic factors but also to shared family environment and behaviours.
2. Poor sleep has consistently been associated with child obesity and has been suggested as an obesity‐related behaviour.

## What this paper adds

1. A longitudinal obesity prevention project in Stockholm provides an opportunity to explore the development of sleep patterns, as well as sleep characteristics, in children at different obesity risks, determined based on parental weight status.
2. Although the development of sleep patterns was similar in children at different obesity risks, unfavourable sleep characteristics, regarding prolonged sleep onset latency, low sleep efficiency and pronounced weekday–weekend variation in sleep schedule, were more common in children at high obesity risk than in children at low obesity risk.

Obesity in parents has been identified as a major risk factor for child obesity. The risk of obesity is considered 4–10 times higher in children with parents suffering from obesity than in children with parents of normal weight.^1^ The transfer of adiposity through generations is not yet fully explained, although it is probably not related solely to genetic factors but also to shared family environment and behaviours.^2^

Increasing evidence has linked insufficient sleep to obesity in both children^3^ and adults.^4^ Delayed and irregular sleep schedules and poor sleep quality have also been related to unhealthy weight, independent of sleep duration, in school‐aged children and adolescents.^5^ Some behaviours have been proposed to contribute to the associations between these unfavourable sleep characteristics and obesity in school‐aged children, such as increased energy intake, emotional eating and more screen time.^6^, ^7^, ^8^ Considered together, these findings suggest poor sleep as an obesity‐related behaviour. The role of parental obesity in several obesity‐related behaviours in children has been studied previously. Although results were mixed, higher dietary fat intake and greater preference for fatty foods and sedentary activities have been observed in children with parents with obesity compared with children with parents of normal weight.^9^, ^10^ This indicates that children with parents suffering from obesity are probably more exposed to an obesogenic family environment, as well as having a genetic predisposition for obesity. However, whether parental obesity is also related to child sleep is less understood. Studies on this are essential for a better understanding of whether sleep patterns are involved in familial vulnerability for obesity development, as well as to provide evidence for or against including sleep in obesity prevention interventions.

The Early Stockholm Obesity Prevention Project (Early STOPP) is a randomised controlled obesity prevention targeting pre‐schoolers with parents with overweight or obesity (at high obesity risk).^11^ Children with parents of normal weight are also included as a low obesity risk group. The aims of Early STOPP are primarily to study whether a family‐based intervention targeting life‐style behaviours (diet, physical activity and sleep pattern) can prevent the development of obesity among children at high obesity risk and, secondarily, to explore the development of these behaviours in children at different obesity risks, as well as the intervention effects on these behaviours.^11^ In the present sub‐study, we aimed to study sleep in children at different obesity risks. We first studied sleep development and sleep characteristics from 1 to 2 years of age in children at different obesity risks. Furthermore, we explored the weekday–weekend sleep variations in children at different obesity risks at 2 years of age, as well as the family factors in relation to the variations.

## Methods

### Study design

Early STOPP is a longitudinal, randomised controlled obesity prevention project. A total of 236 children and families in the Stockholm area were recruited to the project between 2010 and 2013. The study design and recruitment have been previously described in detail.^11^ Briefly, families were recruited randomly when parents visited a local child health‐care centre (CHC) for their child's 8‐month check‐up. The CHCs, financed by the counties, offer routine health check‐ups to children aged 0–6 years free of charge and cover almost all children (99%) in Sweden.^12^ Families with at least one parent with obesity (body mass index (BMI) of at least 30 kg/m^2^), or two parents with overweight (BMI of 25–29.9 kg/m^2^), were recruited to the high obesity risk group (high‐risk) (*n* = 181). Families with two normal‐weight parents (BMI of 18–24.9 kg/m^2^) were recruited to the low obesity risk group (low‐risk) (*n* = 57). Families in the high‐risk group were allocated to either the intervention (*n* = 66) or the control group (*n* = 115) through cluster randomisation of CHCs. No family with underweight parents was recruited in the study. The inclusion criteria were having a child younger 1 year of age, that parents could communicate in Swedish and that the child was without chronic health problems likely to influence growth and development. Annual data collection was performed with children from aged 1 (baseline) to 6 years. The present sub‐study reported on sleep patterns using data from children aged 1–2 years.

Ethical approval was given by the Stockholm Regional Ethical Review Board (2009/217–31), and written informed consent was obtained from all the families. This study is registered at http://clinicaltrials.gov (NCT01198847).

### Intervention

Full details of the intervention programme have been described in the study protocol.^11^ Briefly, the multifaceted intervention, consisting of education and individual target coaching, was aimed at increasing parents' knowledge and skills to promote parenting behaviour regarding child diet, sleep and physical activity. Families in the intervention group received coaching sessions, delivered by a coach trained in motivational interviewing, four times during the first study year (at child age 12, 15, 18 and 21 months) and two times a year during the rest of the study period. The educational material about sleep was adapted for the child's age, based on evidence and best practices, to include information on normal sleep development during the first years of life, ideal environments for promoting self‐regulated sleep habits, practices on keeping consistent sleep schedules, setting bedtime routines and handling night‐time awakenings. The coaching sessions were conducted as an academic detailing process that identified the parents' knowledge deficits about the child sleep and their concerns and worries as a basis for choosing information, which was then tailored to meet the parents' needs.^13^ The families in the control and low‐risk groups received only routine health‐care services from CHCs.

### Participants

**Figure 1:**
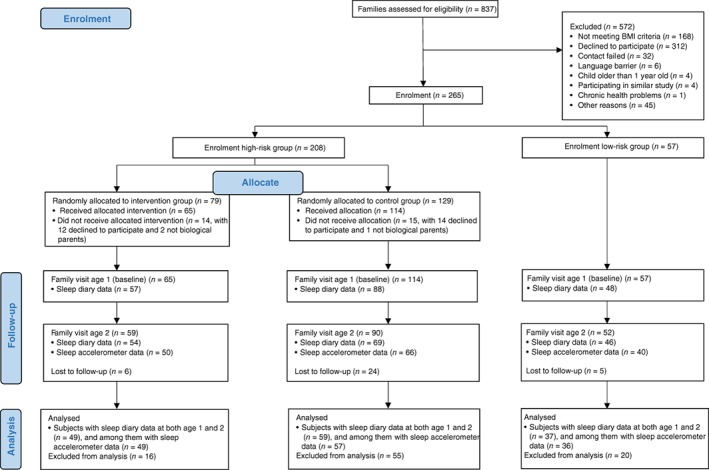
A flowchart of family recruitment in Early Stockholm Obesity Prevention Project and the participants included in the present study.

Of the enrolled 236 children in Early STOPP, 193 and 169 had completed sleep diaries at the 1‐year and 2‐year follow‐up points, respectively. For the present study, 145 children with completed sleep diaries at both time points were included in the analyses to study sleep development and sleep characteristics. Furthermore, among them, 142 with objective sleep data at the 2‐year follow‐up were included in the analysis of the weekday–weekend sleep variations and associated family factors. A flowchart of family recruitment and the participants included in the present sub‐study is presented in Figure 1. Moreover, as no intervention effect was detected on any of the sleep or growth measures at 2 years of age (Table S1, Supporting Information), the analyses in the present study were based on two groups, families with normal‐weight parents (low obesity risk group, *n* = 37) and families with overweight/obese parents (high obesity risk group, *n* = 108), to achieve the study aims.

### Measures

#### Sleep diary

Child sleep at 1 and 2 years of age was followed for 1 week using a parental recorded sleep diary, which is commonly used in child sleep assessment.^14^ The seasons were consistent between assessment points for each individual, which reduced the potential effect on individual sleep schedules of the Scandinavian seasons. The sleep diaries included the following sleep variables: bedtime, sleep onset latency, wake‐up time, nocturnal sleep duration, sleep efficiency, nap time, nap duration and 24‐h total sleep duration. The definitions of the sleep variables are presented in Table 1. The averages of these sleep variables were calculated across the whole week.

**Table 1:** Definitions of sleep variables measured using sleep diary and accelerometer

Sleep diary data		Accelerometer data	
Variables	Definition	Variables	Definition
Bedtime, h:min	Parents recorded the clock time when their child was put to bed at night	Sleep onset, h:min	The clock time for the first of 5 consecutive minutes scored as asleep between 19:00 and 24:00
Sleep onset latency, min	Parents recorded how long it took for the child to fall asleep	Sleep offset, h:min	The clock time for the first of 10 consecutive minutes scored as awake between 04:00 and 10:00
Wake‐up time, h:min	Parents recorded the clock time when their child woke up in the morning	Midpoint of sleep, h:min	Mean clock time between sleep onset and sleep offset
Nocturnal sleep duration, h	Number of minutes between the wake‐up time and bedtime minus the recorded sleep onset latency and wake during sleep period/60	Nocturnal sleep duration, h	Number of minutes scored as sleep between nocturnal sleep onset and sleep offset/60
Sleep efficiency, %	(Nocturnal sleep duration)/(wake‐up time − bedtime)×100	Sleep efficiency, %	(Nocturnal sleep duration)/(sleep offset − sleep onset)×100
Nap time, h:min	Parents recorded the clock time when their child had a nap during the daytime (08:00–19:00)	Onset of nap, h:min	The clock time for the first of 5 consecutive minutes scored as asleep during daytime (08:00–19:00)
Nap duration, h	Parents recorded how many hours the child slept during the daytime (08:00–19:00)	Nap duration, h	Number of minutes scored as asleep during daytime/60
24‐h total sleep duration, h	The total sleep hours during the entire day, including nocturnal sleep duration and daytime naps	24‐h total sleep duration, h	The total sleep hours during the entire day, including nocturnal sleep duration and daytime naps

The upper or lower quartiles of sleep variables generated based on both measuring points were used to classify unfavourable sleep characteristics in terms of bedtime, sleep onset latency, nocturnal sleep duration and sleep efficiency. Children with a bedtime in the upper quartile (later than 20:30) were defined as having ‘late sleep’, and those with sleep onset latency in the upper quartile (longer than 30 min) were defined as having ‘prolonged sleep onset latency’. A nocturnal sleep duration in the lower quartile (shorter than 10 h) was defined as ‘short nocturnal sleep’, and sleep efficiency in the lower quartile (lower than 90%) was defined as ‘low sleep efficiency’. Based on dichotomised unfavourable sleep characteristics across the two measuring points, three trajectories in terms of different sleep variables were defined: (1) ‘never’ (no unfavourable sleep characteristics at any age), (2) ‘transient’ (unfavourable sleep characteristics at either age 1 or age 2 years) and (3) ‘persistent’ (unfavourable sleep characteristics at both age 1 and age 2 years).

#### Accelerometer

At child age 2 years, during the same period as when the sleep diary was recorded, an objective assessment of child sleep was also conducted for seven consecutive days using a wrist‐worn accelerometer, ActiGraph GT3X+ (ActiGraph LLC, Pensacola, FL, USA). The ActiGraph GT3X+ is a tri‐axial accelerometer with a sensitivity of 0.05 g and a sampling rate from 30 to 100 Hz. The activity counts were collected at a 30‐Hz sampling rate and averaged over 1‐s epochs. Using the manufacturer's software ActLife version 6.11.9, the activity data for each minute were scored as either ‘awake’ or ‘asleep’ based on Sadeh's sleep algorithm, which has been validated in children using polysomnography.^15^ Due to the overestimation of night‐time awakenings when using an accelerometer in pre‐school‐aged children,^16^ a secondary algorithm, which has been validated in children aged 2–6 years using both videosomnography and polysomnography,^16^, ^17^ was applied to smooth the data. More specifically, when two or more consecutive minutes with activity counts above 100 were immediately preceded by any activity count above 0, that epoch was scored as the start of an awakening. An awakening was scored as ending at the first of three consecutive minutes with 0 activity counts. Smoothing was automated using an Excel formula (Microsoft, Redmond, WA, USA). Children with at least 4 nights' data were included. A total of 951 nights' data were included in the study, with 716 in the high‐risk group and 229 in the low‐risk group. All actigraphy data were visually reviewed, with references made to sleep diaries and accelerometer logs to identify sleep periods and remove artefacts. The definitions of objectively measured sleep variables are presented in Table 1. Averages of sleep variables across the whole week, weekdays (Sunday to Thursday) and weekends (Friday to Saturday) were calculated. Weekday–weekend sleep variation in different sleep variables were calculated as weekend values minus weekday values.

#### Anthropometry

Trained staff measured the weight and height of both children and parents at child age 1 and 2 years following a standard protocol.^11^ Mean values from three measurements were used in the analyses. BMI was calculated based on weight and height. Based on World Health Organization (WHO) cut‐off points for BMI, parents were categorised as normal weight (18–24.9 kg/m^2^), overweight (25–29.9 kg/m^2^) or obese (≥ 30 kg/m^2^). In children, classification of weight status was based on WHO child growth standards for children aged younger 5 years: normal weight (weight‐for‐height between −2 *z*‐score to 2 *z*‐score), overweight (weight‐for‐height above 2 *z*‐score) or obesity (weight‐for‐height above 3 *z*‐score).^18^ There was no child with weight‐for‐height below −2 *z*‐score.

#### Demographics

Information on child date of birth, gender, living conditions (apartment/terraced house or detached house), if a child attended a day care centre, full‐time (spending ≥30 h/week in day care centres) or part‐time/at home (spending <30 h/week in day care centres), and whether he/she had siblings (yes/no) was collected at baseline using questionnaires. Parental date of birth, education level and ethnic origin (Nordic or not) was also collected. Parents reported their educational levels as compulsory school (9 years), high school (12 years) or college/university. Family education level was defined as low if both parents had attended school for 12 years or less and as high if at least one parent had secondary education, that is, college/university. At the 2‐year follow‐up, information on child living conditions, attending day care centres and having siblings or not was updated. The date when sleep was assessed using an accelerometer was recorded and categorised as summer (from April to September) or winter (from October to March).

### Statistical analysis

Data were presented as means and standard deviations (SD), medians and interquartile ranges or counts (*n*) and percentages (%), depending on their nature. Independent *t*‐tests and *χ* ^2^ tests were used to test the differences in sample characteristics between groups. To compare sleep development from age 1 to 2 years between groups, analysis of variance (ANOVA) for repeated measures was adopted, with age as the within‐subject effect and obesity risk group as the between‐subject effect. The interaction effect between age and group was also tested. Log‐10 transformation was conducted on the variables of sleep onset latency and sleep efficiency to normalise the distributions and fulfil the assumptions for using repeated ANOVA. *χ* ^2^ tests were performed to compare the proportions of the three trajectories of unfavourable sleep characteristics between groups. Then, given the trajectories of unfavourable sleep characteristics, with three categories, multiple logistic regression was performed to confirm the relationships between these characteristics and obesity risk, as well as other family‐related factors, with unfavourable sleep characteristics as dependent variables and obesity risk and other family‐related factors as independent variables.

For the aim of exploring the weekday–weekend sleep variations in children at different obesity risks, independent *t*‐tests were first conducted to compare sleep variations between groups. Furthermore, a multi‐factor ANOVA was performed to identify family factors related to the variations in sleep schedules and sleep duration, with the variations as continual variables and family‐related factors as categorical variables. The weekday–weekend variations in sleep schedules, including sleep onset, sleep offset, midpoint of sleep and nap onset and variations in nocturnal sleep duration, were used as dependent variables. The family obesity risks and other family‐related factors were used as independent variables and included in models simultaneously. Data were analysed using SPSS Statistics, version 21 (IBM, Armonk, NY, USA). All tests were two‐sided, and *P* values of <0.05 were regarded as statistically significant.

## Results

### Participants' characteristics

Among the 236 children in Early STOPP, 145 were included in the present analysis. There was no difference in baseline characteristics between children included and not included (*n* = 91) in the study. However, fathers of not‐included children had a higher BMI than fathers of those included. Moreover, not‐included children had slightly higher weight and BMI than children included at both age 1 and age 2 (Table S2, Supporting Information). Among included children, 37 were in the low‐risk group, and 108 were in the high‐risk group. The two groups of children showed comparable demographic characteristics and growth measurements at both ages. The prevalence of overweight in children increased from age 1 to 2 years in both groups. However, no child with obesity was identified. The only significant difference between groups, apart from parental BMI, was in education, with more parents with a low education level in the high‐risk group (Table 2).

**Table 2:** Characteristics of the study population at age 1 and 2 years in high and low obesity risk groups

		Family obesity risk groups			
		Low risk (*n* = 37)		High risk (*n* = 108)	
		Age 1	Age 2	Age 1	Age 2
Characteristics					
Child, *n* (%)					
Gender	Boy	15 (41.0)	—	56 (51.8)	—
Having siblings	Yes	19 (51.4)	23 (62.2)	50 (46.3)	62 (59.0)
Attending day care	Yes, full time	1 (2.7)	31 (83.8)	2 (1.9)	91 (84.2)
Mother					
Age, years, mean (SD)		33.3 (4.2)	34.3 (4.2)	33.4 (4.5)	34.4 (4.5)
BMI, kg/m^2^, mean (SD)		22.5 (2.1)	23.0 (2.2)	32.2 (6.4)**	31.9 (6.5)**
Education, *n* (%)	≤12 years of school	8 (21.6)	—	44 (41.1)*	—
Ethnicity, *n* (%)	Other than Nordic	5 (13.5)	—	7 (6.5)	—
Father					
Age, years, mean (SD)		35.7 (4.9)	36.7 (4.9)	35.3 (5.0)	36.3 (5.0)
BMI, kg/m^2^, mean (SD)		23.0 (1.6)	23.3 (1.6)	29.3 (4.2)**	28.9 (6.7)**
Education, *n* (%)	≤12 years of school	9 (25.0)	—	50 (50.5)*	—
Ethnicity, *n* (%)	Other than Nordic	2 (5.4)	—	12 (11.9)	—
Family, *n* (%)					
Education level†	Low	4 (10.8)	—	30 (28.8)*	—
Living conditions	Apartment	20 (54.1)	16 (43.2)	52 (48.1)	48 (44.4)
Child measurements					
Weight, kg, mean (SD)		9.9 (1.0)	12.7 (1.2)	10.1 (1.0)	13.2 (1.4)
Height, cm, mean (SD)		75.8 (2.7)	87.2 (3.0)	76.2 (2.5)	88.0 (2.9)
BMI, kg/m^2^, mean (SD)		17.4 (1.3)	16.7 (1.4)	17.3 (1.4)	17.0 (1.3)
Overweight, *n* (%)	Yes	2 (5.4)	4 (10.8)	5 (4.6)	13 (12.5)

* *P* < 0.05.

** *P* < 0.01.

† Family education level: Low level = neither parent's education >12 years, high level = at least one parent's education >12 years.

Missing data: Child having siblings at age 2 (*n* = 3), child growth measurements at age 2 (*n* = 4), maternal education level (*n* = 1), maternal ethnicity (*n* = 1), paternal education level (*n* = 10), paternal ethnicity (*n* = 7), family education level (*n* = 4).

Difference in variable between low‐ and high‐risk groups was detected at the child's same age level.

—, The information was only collected at the age 1 (baseline). BMI, body mass index; SD, standard deviation.

### Sleep development and characteristics from age 1 to age 2

#### Sleep development

Of the parental reported sleep, at age 1, only six (4%) children's sleep duration did not reach the amount suggested in the sleep recommendation,^19^ with sleep shorter than 11 h per day; one of them was in the low‐risk group and the rest in the high‐risk group. The number of children who did not reach the recommended level had increased to 28 (19%) at age 2, with 10 in the low‐risk group and 18 in the high‐risk group. As can be seen in Table 3, age differences of child sleep patterns were detected in all sleep variables, except sleep efficiency. Compared with children at age 1, children at age 2 had a later bedtime and earlier wake‐up time, and it took a slightly longer time for them to fall asleep. Sleep duration decreased from age 1 to age 2, with a large reduction in nap duration. Group differences could only be detected in sleep onset latency, showing that children in the high‐risk group had longer sleep onset latency than children in the low‐risk group at both ages. However, the medians of sleep latency for the two groups at age 2 differed by only 1 min, and the effect size of the difference was relatively small (Cohen's effect size 0.12). Moreover, there was no group difference in other sleep variables. Furthermore, no statistically significant interaction effect between age and group could be detected, indicating that there was no difference in the sleep development between children from different groups.

**Table 3:** Average sleep patterns across ages among children in different obesity risk groups, based on sleep diary data

	Low‐risk group		High‐risk group		Age effect, *P* value	Group effect, *P* value	Age × group effect, *P* value
	Age 1	Age 2	Age 1	Age 2			
Bedtime, h:min, mean (SD)	20:00 (46)	20:17 (30)	20:11 (52)	20:16 (42)	0.008	0.51	0.14
Sleep onset latency, min, median (q1, q3)	13 (6, 22)	22 (15, 28)	21 (14, 28)	23 (15, 35)	0.003	0.01	0.08
Wake‐up time, h:min, mean (SD)	06:49 (41)	06:36 (30)	06:59 (42)	06:46 (41)	0.001	0.16	0.93
Nocturnal sleep duration, h, mean (SD)	10.6 (0.7)	10.2 (0.7)	10.5 (0.8)	10.3 (0.7)	<0.001	0.87	0.09
Sleep efficiency, %, median (q1, q3)	94 (92, 96)	93 (92, 95)	92 (89, 94)	93 (91, 95)	0.12	0.06	0.10
Nap duration, h, mean (SD)	2.0 (0.6)	1.3 (0.5)	2.0 (0.5)	1.2 (0.5)	<0.001	0.83	0.75
24‐h total sleep duration, h, mean (SD)	12.6 (0.7)	11.4 (0.7)	12.4 (0.7)	11.6 (0.7)	<0.001	0.83	0.05

Analysis of variance (ANOVA) for repeated measures was conducted. Log‐10 transformation was conducted on non‐normally distributed data to normalise the data distributions and fulfil the assumptions for using ANOVA for repeated measures.

SD, standard deviation.

#### Unfavourable sleep characteristics

**Figure 2:**
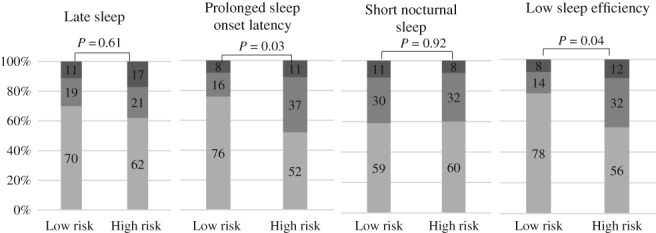
Proportions of unfavourable sleep characteristics trajectories (never, transient and persistent) in children from different obesity risk groups, in terms of late sleep, prolonged sleep onset latency, short nocturnal sleep and low sleep efficiency (%), based on sleep diary data. (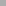), Never (no unfavourable sleep characteristics at any age); (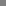), transient (unfavourable sleep characteristics at either age 1 or age 2); (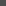), persistent (unfavourable sleep characteristics at both age 1 and age 2); sleep variables are based on sleep diary data.

Based on sleep diary data, most of the children did not show unfavourable sleep characteristics or only had transient unfavourable sleep characteristics. About 10% of children had persistent unfavourable sleep characteristics for certain sleep variables (Fig. 2). Children in the high‐risk group showed unfavourable sleep characteristics regarding prolonged sleep onset latency and lower sleep efficiency to a greater extent than children in the low‐risk group. After adjusting for family confounders, the differences were confirmed. Compared with children in the low‐risk group, the children in the high‐risk group were more likely to experience transient prolonged sleep onset latency (odds ratio (OR) = 3.8, 95% confidence interval (CI) 1.4–11.1, *P* = 0.008), as well as transient lower sleep efficiency (OR = 4.0, 95% CI: 1.3–12.5, *P* = 0.01). Moreover, we found that, compared with girls, boys were more likely to experience persistent short sleep duration (OR = 10.6, 95% CI: 2.0–55.9, *P* = 0.005), as well as transient lower sleep efficiency (OR = 2.6, 95% CI: 1.1–6.3, *P* = 0.02).

We also explored if children with transient or persistent unfavourable sleep characteristics gained more weight from age 1 to age 2. After adjusting for obesity risk, child gender and BMI at age 1, there was no significant difference in BMI at age 2 among children with different sleep trajectories (data not shown).

### Weekday–weekend sleep at age 2

#### Weekday–weekend sleep variations

The objectively measured weekday–weekend sleep patterns and variations in children from different groups are presented in Table 4. Children in both groups showed delayed sleep schedules and slightly longer sleep duration on weekends compared to weekdays. There was no difference in weekday–weekend variation in sleep duration between groups. However, compared with children in the low‐risk group, children in the high‐risk group showed more delayed sleep offset, midpoint of sleep and nap onset during weekends than weekdays, indicating that children from high‐risk families had more weekday–weekend variation in sleep schedules.

**Table 4:** Weekday–weekend (WD–WE) sleep patterns and variations in children at age 2 in different obesity risk groups based on accelerometer data

	Family obesity risk						*P* values§
	Low‐risk group			High‐risk group			
	Weekday	Weekend	WD–WE (min)†	Weekday	Weekend	WD–WE (min)†	
Sleep onset, h:min, mean (SD)	20:32 (61)	20:39 (58)	6 (15)	20:25 (56)	20:41 (66)	18 (36)	0.21
Sleep offset, h:min, mean (SD)	06:34 (53)	06:40 (56)	6 (36)	06:32 (49)	06:58 (60)	24 (36)	0.003
Midpoint of sleep, h:min, mean (SD)	01:33 (54)	01:39 (52)	6 (24)	01:28 (48)	01:50 (57)	20 (24)	0.007
Nocturnal sleep duration, h, mean (SD)	9.1 (0.7)	9.2 (0.7)	7 (37)	9.2 (0.7)	9.4 (0.8)	11 (42)	0.63
Sleep efficiency, %, mean (SD)	88.2 (4.2)	89.8 (4.6)	1.6 (4.4)	87.9 (4.8)	88.6 (4.7)	0.6 (5.0)	0.27
Onset of nap, h:min, mean (SD)	12:12 (55)	12:17 (72)	7 (84)	12:13 (43)	13:00 (73)	48 (72)	0.006
Nap duration, h, mean (SD)	1.3 (0.3)	1.5 (0.7)	11 (36)	1.3 (0.4)	1.4 (0.5)	5 (29)	0.34
24‐h total sleep duration, h, mean (SD)	10.4 (0.6)	10.7 (0.9)	12 (48)	10.5 (0.7)	10.7 (0.8)	12 (49)	0.97

† Weekday–weekend, weekday and weekend sleep variations were calculated using: Sleep variables (weekend) minus sleep variables (weekday).

§ *P* values for the comparisons of weekday–weekend sleep variations between children in the high‐ and low‐risk groups, based on independent *t*‐tests.

SD, standard deviation.

#### Family factors associated with weekday–weekend sleep variations

After adjustment for other family‐related factors, the multivariate analysis confirmed that children in the high‐risk group had more weekday–weekend variation regarding sleep offset, midpoint of sleep and nap onset than children in the low‐risk group (Table 5). Family education level was also associated with weekday–weekend sleep variations, with children from families with low education having more delayed sleep offset and midpoint of sleep on weekends compared with children from families with high education. In addition, girls and children with siblings showed more weekday–weekend variation in sleep onset than their counterparts, respectively. However, no association between variation in sleep duration and other family factors could be identified, nor could any associations between weekday–weekend sleep variations and child weight status.

**Table 5:** Multivariate analysis of weekday–weekend (WD–WE) sleep variation and associated family factors based on accelerometer data

	WD‐WE, min, effect estimate (adjusted mean difference, 95% CI)†				
	Sleep onset	Sleep offset	Midpoint of sleep	Nap onset	Nocturnal sleep duration
Family obesity risk					
High risk	10 (−5, 24)	18 (4–33)*	14 (3–25)*	42 (10–74)**	4 (−13, 20)
Low risk	0 (ref)	0 (ref)	0 (ref)	0 (ref)	0 (ref)
Family education level					
Low level	2 (−15, 19)	11 (5–27)*	7 (6–20)*	−32 (−67, 5)	9 (−10, 28)
High level	0 (ref)	0 (ref)	0 (ref)	0 (ref)	0 (ref)
Attending day care					
Part time or at home	−11 (−28, 7)	−8 (−25, 8)	−9 (−22, 4)	−28 (−66, 10)	2 (−18, 21)
Full time	0 (ref)	0 (ref)	0 (ref)	0 (ref)	0 (ref)
Having siblings					
Yes	16 (2–30)*	5 (−8, 19)	11 (−1, 21)	19 (−11, 49)	−3 (−19, 14)
No	0 (ref)	0 (ref)	0 (ref)	0 (ref)	0 (ref)
Living condition					
Apartment	−6 (−20, 8)	20 (−6, 32)	7 (−4, 17)	22 (−7, 53)	9 (−7, 25)
Terraced or detached house	0 (ref)	0 (ref)	0 (ref)	0 (ref)	0 (ref)
Gender					
Boy	−15(−28, 2)*	5 (−8, 18)	−5 (−15, 5)	−12 (−42, 17)	13 (−3, 28)
Girl	0 (ref)	0 (ref)	0 (ref)	0 (ref)	0 (ref)
Weight status					
Overweight	18 (−7, 42)	−15 (−39, 8)	2 (−17, 20)	−5 (−64, 53)	−19 (−47, 8)
Normal weight	0 (ref)	0 (ref)	0 (ref)	0 (ref)	0 (ref)
Season					
Summer	−4 (−17, 10)	−7 (−19, 7)	5 (−5, 15)	−13 (−42, 15)	7 (−8, 22)
Winter	0 (ref)	0 (ref)	0 (ref)	0 (ref)	0 (ref)

* *P* < 0.05.

** *P* < 0.01.

† Weekday–weekend sleep variation was calculated using: Sleep variables (weekend)–sleep variables (weekday).

Multivariate analysis of the family factors associated with weekday–weekend variation in sleep schedules, regarding sleep onset, sleep offset, midpoint of sleep, nap onset and nocturnal sleep duration, were conducted using analysis of variance.

CI, confidence interval; ref, reference.

## Discussion

We observed that unfavourable sleep characteristics regarding prolonged sleep onset latency and low sleep efficiency were more common in children at high obesity risk than in children at low obesity risk. We also found that more weekday–weekend variation in sleep schedules of children was associated with high obesity risk. Considered together, these results may reflect the unfavourable sleep hygiene and inconsistent sleep routines in families at high obesity risk.

The development of sleep patterns was similar in both groups. Although the children at high obesity risk had slightly longer sleep onset latency than children at low risk, the effect size of the statistically significant difference was relatively small, indicating that the clinical relevance of the prolonged sleep onset latency is probably low. However, we still observed that children at high obesity risk were more likely to suffer from unfavourable sleep characteristics like difficulty falling asleep and disturbed nocturnal sleep. The persistence of these unfavourable sleep characteristics may reflect more sleep problems in children with obese parents. It has been reported that approximately 25–50% of pre‐schoolers experience some type of sleep problem at some point during childhood, mainly difficulties falling asleep and frequent nocturnal awakenings.^20^ Family is one of the essential contexts for child sleep. Family and parental factors, such as low socio‐economic status, emotion disorders and high involvement during child sleep, have been associated with poor sleep and more sleep problems in children.^21^, ^22^ The association between child and parental sleep quality has also been observed previously.^23^, ^24^ Our finding added that family high obesity risk, based on parental weight, was probably also related to child poor sleep characteristics. Given the association between poor sleep and unhealthy weight development,^3^ the prevalent unfavourable sleep characteristics in children with obese parents may also contribute to the transfer of adiposity through generations in these high‐risk families. However, in the present study, no association between these unfavourable sleep characteristics and children's weight could be identified as early as 2 years of age. It may reflect that the impact of poor sleep characteristics on weight development in children is a long‐term effect. Therefore, the associations between these sleep characteristics and children's weight will be followed in our future studies in order to clarify the role of sleep in the transfer of adiposity from parents to their children.

Variation is also an important aspect of individual sleep.^25^ The pronounced weekday–weekend variation has been identified as a proxy for circadian misalignment and has been associated with chronic sleep loss, increased BMI and emotion disorders in adolescents and adults.^26^, ^27^ Weekday–weekend variation is less prominent in young children than in adolescents and adults because of the prevalent morning chronotype as well as fewer social demands.^28^ In our analysis, we found that large weekday–weekend variation in sleep schedules of young children was associated with high obesity risk, probably indicating that not all children were at equal risk of sleep variation. Children with parents suffering from obesity showed a larger shift towards later sleep schedules during the weekend than children with normal‐weight parents. While the absolute differences were small, they may indicate an early start of irregular sleep habits in these high‐risk families. They may also reflect these children's biological preference for an evening chronotype, which is related to early weekday–weekend shifts in sleep timing.^29^ We also found that weekday–weekend variation in sleep schedules was associated with family education level. Parents with low education have been reported as having less regular sleep practices than parents with higher education.^30^ Given that obesity is also more prevalent among people with low socio‐economic status, the regularity of sleep routines may require more attention in children from families where parents are obese and low‐educated.

The findings of differential sleep patterns in children at different obesity risks have some resonance with findings in individuals with obesity. It has also been reported that adolescents and adults suffering from obesity tend to have poorer sleep quality, later sleep and larger weekday–weekend sleep variation than individuals of normal weight.^31^, ^32^, ^33^ In our study, the prevalence of overweight was equal in children at different obesity risks. Therefore, the observed unfavourable sleep characteristics and greater weekday–weekend variations in children at high obesity risk were probably not directly related to child weight. Instead, they may, to a certain extent, reflect some neurophysiological characteristics of children with parents suffering from obesity and may also suggest that those children are more exposed to unfavourable sleep hygiene and inconsistent sleep schedules shaped by their parents. Thus, from the perspective of obesity prevention, regular sleep routines may need to be emphasised more in high‐risk families. However, in our study, no intervention effect can be identified yet on either child sleep or weight development after 1 year's intervention.

The sleep data in our study were collected using both subjective and objective methods. Accelerometer sleep schedules data were correlated with sleep schedules data collected using diaries (*r* = 0.78–0.82) and the absolute discrepancy averaged 25 min for sleep onset and 11 min for sleep offset. Moreover, accelerometer nocturnal sleep duration was 66 min shorter on average than the duration collected using a sleep diary, with correlation *r* = 0.63. Accelerometer sleep efficiency was 5% lower on average than sleep efficiency calculated using sleep diaries, with correlation *r* = 0.21. Data collected using diaries showed acceptable agreement with objectively measured data. However, a large discrepancy between subjectively reported and accelerometer‐estimated sleep duration was found, as well as a weak correlation between subjective and objective sleep efficiency. These findings were in line with a number of prior studies^34^, ^35^, ^36^ and probably reflect that parents have only limited awareness of children's sleep behaviours during the night, such as night awakenings.^14^, ^36^

Some limitations should be addressed. The results of longitudinal sleep development and characteristics were based on sleep diary data. The limitations of sleep diaries, such as recall bias and low validity of sleep quality evaluation, may result in some inaccuracies in our results. Moreover, given the qualitative differences in definitions of subjective and objective measurements, as well as the marked discrepancies between subjectively and objectively estimated sleep duration and efficiency, we considered that it was probably not appropriate to classify unfavourable sleep characteristics in our data by using cut‐off values from the sleep quality recommendation published by National Sleep Foundation,^37^ which was based on studies using objective measurements. Instead, we chose quartiles to classify sleep characteristics. While it may be somewhat arbitrary, the overall prevalence of unfavourable sleep characteristics in our study was in line with previous reports which have reported that 25–50% toddlers have experienced some sleep problems during the first years of life.^20^ Parents' own sleep discrepancies between weekday and weekend, which were not included in this study, may partially explain child weekday–weekend sleep variation.^28^ Furthermore, participants in the Early STOPP had higher education levels than the general population,^38^ which might impact the generalisability of study results. In addition, the size of the population, as well as the differential sample size in the two groups with a small number of children at low obesity risk, might limit the possibility of detecting small but statistically significant differences, and the statistical analyses were performed without controlling for multiple comparisons, which may increase the risk of type I errors. Possible other confounders, which were not adjusted for in this study, should also be included in future studies. Finally, no conclusion on causation of family obesity risk and child sleep variability can be drawn because of the cross‐sectional analysis of this part of data.

## Conclusions

Children at high obesity risk were more likely to experience unfavourable sleep characteristics, such as prolonged sleep onset latency, low sleep efficiency and pronounced weekday–weekend variation in sleep schedules, than children at low obesity risk. Although the differences were relatively small, they may reflect the unfavourable sleep hygiene and inconsistent sleep routines in families at high obesity risk. Further studies are needed to clarify whether these unfavourable sleep characteristics in the long run contribute to the progression of obesity in children with overweight/obese parents.

## Supporting information


**Table S1**. Characteristics, growth measurements and sleep diary data of the study population at age 2 in low‐risk, control and intervention groups.

**Table S2**. Baseline characteristics and measurements between children included and not included in the present analysis.
