# Supplementary material for: Development of sleep patterns in children with obese and normal‐weight parents
Source: J Paediatr Child Health. 2018 Nov 10;55(7):809–18. doi: 10.1111/jpc.14294 (PMC6899924; doi:10.1111/jpc.14294)
Supplement: Supplementary file 1 — Table S1. Characteristics, growth measurements and sleep diary data of the study population at age 2 in low‐risk, control and intervention groups. [file JPC-55-809-s001.docx]

Supplementary 1. Characteristics, growth measurements and sleep diary data of the study population at age 2, in low-risk, control and intervention groups.

|  |  | Low-risk |  | Control |  | Intervention |
| --- | --- | --- | --- | --- | --- | --- |
|  |  | n = 37 |  | n = 59 |  | n = 49 |
| **Characteristics** |  |  |  |  |  |  |
| **Child** |  |  |  |  |  |  |
| Gender, n (%) | Boy | 15 (41.0) |  | 31 (52.5) |  | 25 (51.0) |
| Having siblings, n (%) | Yes | 23 (62.2) |  | 35 (59.3) |  | 27 (58.7) |
| Attending day care, n (%) | Yes, full-time | 31 (83.8) |  | 51 (86.4) |  | 40 (81.6) |
| **Mother** |  |  |  |  |  |  |
| BMI, kg/m^2^ |  | 23.0 (2.2) |  | 31.4 (5.6) |  | 32.3 (7.6) |
| Education, n (%) | ≤ 12 years of school | 8 (21.6) |  | 23 (39.0) |  | 21 (43.8) |
| Ethnicity, n (%) | Other than Nordic | 5 (13.5) |  | 4 (6.8) |  | 3 (6.3) |
| **Father** |  |  |  |  |  |  |
| BMI, kg/m^2^ |  | 23.3 (1.6) |  | 29.8 (4.3) |  | 28.8 (4.2) |
| Education, n (%) | ≤ 12 years of school | 9 (25.0) |  | 29 (52.7) |  | 21 (47.7) |
| Ethnicity, n (%) | Other than Nordic | 2 (5.4) |  | 7 (12.5) |  | 5 (11.1) |
| **Family** |  |  |  |  |  |  |
| Education level^†^, n (%) | Low | 4 (10.8) |  | 19 (33.3) |  | 11 (23.4) |
| Living conditions, n (%) | Apartment | 16 (43.2) |  | 22 (37.3) |  | 26 (53.1) |
| **Growth measurements** | |  |  |  |  |  |
| Weight, kg |  | 12.7 (1.2) |  | 13.3 (1.4) |  | 12.9 (1.4) |
| Height, cm |  | 87.2 (3.0) |  | 88.2 (2.9) |  | 87.7 (2.9) |
| BMI, kg/m^2^ |  | 16.7 (1.4) |  | 17.1 (1.4) |  | 16.8 (1.2) |
| Overweight, n (%) |  | 4 (10.8) |  | 10 (17.5) |  | 3 (6.4) |
| **Sleep diary data** | |  |  |  |  |  |
| Bedtime (h:min) |  | 20:17 (30) |  | 20:13 (40) |  | 20:17 (45) |
| Sleep onset latency^§^ (min) |  | 22 (15, 28) |  | 23 (16, 32) |  | 25 (14, 38) |
| Wake-up time (h:min) |  | 06:36 (30) |  | 06:45 (39) |  | 06:44 (42) |
| Nocturnal sleep duration (h) |  | 10.2 (0.7) |  | 10.4 (0.6) |  | 10.3 (0.7) |
| Sleep efficiency^§^ (%) |  | 93 (92, 95) |  | 93 (92, 96) |  | 93 (90, 95) |
| 24-h total sleep duration (h) |  | 11.4 (0.7) |  | 11.6 (0.7) |  | 11.5 (0.7) |

Values are means (SD), unless otherwise indicated. Mean (SD) for continuous variables; n (%) for categorical data; ^§^Median (q1, q3) for non-normally distributed data, sleep onset latency and sleep efficiency.

BMI = Body mass index, h = hour, min = minute.

^†^ Family education level: low level = neither parent’s education > 12 years, high level = at least one parent’s education > 12 years.

Independent t-tests and chi-square tests were performed between control and intervention groups and no difference in characteristics, growth measurements or sleep diary data could be detected.

Missing data: child having siblings at age 2 (n = 3), maternal education level (n = 1), maternal ethnicity (n = 1), paternal education level (n = 10), paternal ethnicity (n = 7), family education level (n = 4).
