# Supplementary material for: Development of sleep patterns in children with obese and normal‐weight parents
Source: J Paediatr Child Health. 2018 Nov 10;55(7):809–18. doi: 10.1111/jpc.14294 (PMC6899924; doi:10.1111/jpc.14294)
Supplement: Supplementary file 2 — Table S2. Baseline characteristics and measurements between children included and not included in the present analysis. [file JPC-55-809-s002.docx]

Supplementary 2. Baseline characteristics and measurements between children included and not included in the present analysis.

|  |  | Included |  | Not included | P value |
| --- | --- | --- | --- | --- | --- |
|  |  | n = 145 |  | n = 91 |  |
| **Baseline characteristics** |  |  |  |  |  |
| **Child** |  |  |  |  |  |
| Gender, n (%) | Boy | 71 (49.0) |  | 42 (46.2) | 0.68 |
| Having siblings, n (%) | Yes | 69 (48.6) |  | 35 (43.8) | 0.75 |
| Attending day care, n (%) | Yes, full-time | 3 (2.0) |  | 2 (2.5) | 0.67 |
| **Mother** |  |  |  |  |  |
| BMI, kg/m^2^ |  | 29.7 (7.1) |  | 29.3 (6.4) | 0.65 |
| Education, n (%) | ≤ 12 years of school | 52 (36.1) |  | 32 (39.5) | 0.58 |
| **Father** |  |  |  |  |  |
| BMI, kg/m^2^ |  | 27.6 (4.7) |  | 29.5 (5.6) | 0.01 |
| Education, n (%) | ≤ 12 years of school | 59 (43.7) |  | 34 (47.2) | 0.62 |
| **Family** |  |  |  |  |  |
| Education level^†^, n (%) | Low | 34 (24.1) |  | 17 (22.4) | 0.96 |
| Living conditions, n (%) | Apartment | 72 (49.7) |  | 44 (56.4) | 0.47 |
| **Child measurements** |  |  |  |  |  |
| Weight, kg | Age 1 | 10.1 (1.1) |  | 10.5 (1.4) | 0.02 |
|  | Age 2 | 13.0 (1.4) |  | 13.6 (2.1) | 0.05 |
| Height, cm | Age 1 | 76.1 (2.9) |  | 76.6 (3.2) | 0.19 |
|  | Age 2 | 87.8 (2.9) |  | 88.3 (4.3) | 0.43 |
| BMI, kg/m^2^ | Age 1 | 17.3 (1.3) |  | 17.8 (1.7) | 0.03 |
|  | Age 2 | 16.9 (1.3) |  | 17.5 (1.8) | 0.03 |
| Overweight/obesity, n (%) | Age 1 | 7 (4.8) |  | 11 (12.4) | 0.04 |
|  | Age 2 | 17 (12.1) |  | 8 (14.5) | 0.70 |

Values are means (SD), unless otherwise indicated.

Mean (SD) for continuous variables; n (%) for categorical data.

p values: Independent t-tests and chi-square tests were performed, respectively.

^†^ Family education level: low level = neither parent’s education > 12 years, high level = at least one parent’s education > 12 years.

Missing data in children included in the present study: maternal education level (n = 1), paternal education level (n = 10), family education level (n = 4), child measurements at age 2 (n = 4).

Missing data in children not-included in the present study: child having siblings (n = 11), attending day care centre (n = 11), maternal education level (n = 10), paternal education level (n = 19), family education level (n = 15), living conditions (n = 13), child measurements at age 1 (n = 2) and at age 2 (n = 36).
